# Case Report: Neonatal ultra-severe hemolytic anemia complicated by pulmonary hypertension induced by compound heterozygous variants in the *KLF1* gene and literature review

**DOI:** 10.3389/fped.2026.1943478

**Published:** 2026-10-13

**Authors:** Yunzhi Liu, Dejian Yuan, Yanxing Lv, Biwen Huang, Lanqiu Hu, Yongjiang Jiang

**Affiliations:** 1 Department of Neonatology, Liuzhou Hospital, Guangzhou Women and Children's Medical Center, Liuzhou, Guangxi, China; 2 Department of Genetics and Eugenics, Liuzhou Hospital, Guangzhou Women and Children's Medical Center, Liuzhou, Guangxi, China

**Keywords:** case report, compound heterozygous variant, congenital dyserythropoietic anemia, *KLF1* gene, neonatal anemia, pulmonary hypertension

## Abstract

**Objective:**

To investigate the clinical characteristics, genotypic profiles, and diagnostic/therapeutic strategies for congenital hemolytic anemia induced by compound heterozygous variants in the *KLF1* gene.

**Methods:**

We retrospectively analyzed the clinical data of a neonate who presented primarily with ultra-severe anemia, elevated direct bilirubin, and pulmonary hypertension. Whole-exome sequencing (WES) was utilized for variant identification, followed by a comprehensive review of literature.

**Results:**

The patient, a female neonate, developed severe anemia within 10 h of birth, accompanied by rapidly progressing hyperbilirubinemia (predominantly direct hyperbilirubinemia). Bedside echocardiography revealed a patent ductus arteriosus (PDA) and pulmonary hypertension. Trio-WES identified pathogenic compound heterozygous variants in the *KLF1* gene: c.519_525dup (p.Gly176ArgfsTer179) and c.1012C > A (p.Pro338Thr). Following multiple red blood cell transfusions and mechanical ventilatory support, the infant's condition stabilized. During a 24-month follow-up, the patient exhibited transfusion-dependent anemia.

**Conclusion:**

For neonates presenting with unexplained (Coombs/DAT-negative) severe hemolytic anemia, particularly when complicated by pulmonary hypertension and atypical cholestasis, early genetic testing should be performed to rule out *KLF1*-related diseases.

## 1 Introduction

Neonatal hemolytic anemia represents a high-risk neonatal hematological emergency. Common etiologies include immune-mediated hemolysis, perinatal infection, red-cell membrane defects, enzyme deficiencies, and globin-gene disorders (1). A subset of unexplained Coombs-negative severe hemolytic anemia can be attributed to pathogenic variants in *KLF1*, encoding Krüppel-like factor 1, a master transcriptional regulator of erythropoiesis (2). *KLF1* governs erythroid progenitor differentiation, hemoglobin synthesis, and perinatal globin-gene switching from fetal hemoglobin (HbF) to adult hemoglobin (HbA); loss-of-function variants disrupt erythroid maturation and produce congenital dyserythropoietic anemia (CDA) type IV (2, 3).

The phenotypic spectrum of *KLF1*-related disease is markedly heterogeneous, ranging from asymptomatic hereditary persistence of fetal hemoglobin (HPFH) to life-threatening prenatal non-immune hydrops fetalis, severe early-neonatal hemolysis, and childhood chronic transfusion-dependent anemia (4–6). Neonatal cases driven by compound-heterozygous *KLF1* pathogenic variants are particularly severe and frequently complicated by pulmonary hypertension and atypical cholestatic hyperbilirubinemia, yet these manifestations remain under-recognized in routine neonatal practice (5, 7).

Most existing literature consists of scattered single-case reports; large case series are limited, and the pathophysiological links among severe anemia, pulmonary hypertension, and direct hyperbilirubinemia in affected neonates require further clarification (3, 8). In this article, we describe a neonate with ultra-severe non-immune hemolytic anemia, pulmonary hypertension, and conjugated hyperbilirubinemia due to compound heterozygous *KLF1* variants. We also perform a narrative literature review to summarize clinical features, diagnostic pitfalls, and management strategies, aiming to raise clinicians’ awareness of this rare genetic disorder.

## 2 Case presentation

### 2.1 Clinical data

The patient was a female neonate, aged 10 h, born to a G2P2 mother at a gestational age of 37 ^+^ ^6^weeks, with a birth weight of 2,810 g. She was delivered via cesarean section due to “fetal distress and a scarred uterus.” The amniotic fluid was grade II turbid (volume approx. 800 mL); the placenta and umbilical cord showed no abnormalities. Apgar scores were 8 at 1 min, 9 at 5 min, and 9 at 10 min.

Immediately after birth, the neonate exhibited extreme, generalized pallor of the skin and mucous membranes, rapidly followed by scattered ecchymoses and respiratory distress. After receiving invasive respiratory support and red blood cell transfusions at an external hospital, she was transferred to our Neonatal Intensive Care Unit (NICU) due to the critical nature of her illness. The parents were non-consanguineous and denied any family history of hereditary diseases. The patient's 4-year-old sister was healthy.

Upon admission, the patient was conscious under mechanical ventilation via endotracheal intubation, showing marked cutaneous and mucosal pallor. Scattered ecchymoses were visible on the face, chest, and abdomen, with extensive purpura and petechiae noted on the back. Chest expansion was bilaterally symmetrical, and breath sounds were coarse in both lungs without dry or moist rales. Her heart rate was 154 beats/min with a regular rhythm and crisp heart sounds; no pathological murmurs were heard in any valvular auscultation zone. The abdomen was soft and non-distended; the liver was enlarged and palpable 1 cm below the umbilical line, while the spleen was not palpable. Bowel sounds were hypoactive. Muscle tone in all four limbs was normal, and the capillary refill time was 2 s. Pre-ductal (right upper limb) oxygen saturation (SpO_2_) was 99%, while post-ductal (lower limb) saturation was 86%.

### 2.2 Laboratory and ancillary investigations

A complete blood count (CBC) performed immediately after birth at the referral hospital showed a hemoglobin (Hb) level of 73 g/L (145–220 g/) and a red blood cell (RBC) count of 1.67 × 10^12^/L (4.7–6.5 × 10^12^/L). Upon admission to our hospital, a repeat CBC showed an Hb level of 83 g/L (145–220 g/), RBC count of 2.89 × 10^12^/L (4.7–6.5 × 10^12^/L), mean corpuscular volume (MCV) of 91.3 fL (95–110 fL), mean corpuscular hemoglobin (MCH) of 28.7 pg (31–37 pg), mean corpuscular hemoglobin concentration (MCHC) of 314 g/L (320–360 g/L), and a platelet count of 153 × 10⁹/L (150–400 × 10⁹/L).

Serial laboratory measurements were collected and summarized in Table 1. Biochemical analysis at 12 h of life showed total bilirubin (TBIL) 273.5 μmol/L, direct bilirubin (DBIL) 76.7 μmol/L, indirect bilirubin (IBIL) 196.8 μmol/L, and aspartate aminotransferase (AST) 667 U/L. At 24 h after birth, TBIL reached 288.8 μmol/L with DBIL 97.9 μmol/L; at 48 h, TBIL was 326.5 μmol/L and DBIL rose to 185.6*μ*mol/L. Serial values demonstrated a progressive shift toward predominantly direct hyperbilirubinemia after 48 h of life.

**Table 1:** Serial biochemical laboratory findings of the patient.

Post-natal time point	TBIL (μmol/L)	DBIL (μmol/L)	ALT (U/L)	TBA (μmol/L)	GGT (U/L)
12 h	273.5	76.7	24	12.8	52
24 h	288.8	97.9	—	—	—
48 h	326.5	185.6	—	—	—
60 h	303.6	216.7	23	22	39
6 d	183.4	145.6	25	32.8	69
13 d	209.2	183.3	116	56.9	106
20 d	194.9	175.5	269	55.7	104
1 M	85.2	80.6	155	82.1	145
1.5 M	58.7	51.2	71	53	89

Reference intervals: direct bilirubin (DBIL), <17 μmol/L; alanine aminotransferase (ALT), 6–44 U/L; total bile acid (TBA), <10 μmol/L; gamma-glutamyl transferase (GGT), 9–150 U/L. Total bilirubin (TBIL) reference ranges vary according to post-natal age. Reference ranges may vary across different laboratories and analytical platforms. An em dash (—) indicates data not available.

Regarding specialized workups: both maternal and neonatal blood types were O-positive (Rh+), and both the direct antiglobulin test (DAT/Coombs) and screening for hemolytic disease of the newborn (HDN) were negative. Thyroid function, cytomegalovirus (CMV) screening, and neonatal tandem mass spectrometry screening yielded no abnormalities. Neonatal screening returned a positive result for thalassemia; however, thalassemia workups for both parents and a thalassemia gene panel for the patient revealed no abnormalities.

Imaging: Bedside echocardiography demonstrated a patent ductus arteriosus (PDA, diameter 7.0 mm) with a right-to-left shunt at the great artery level, and pulmonary artery hypertension (PAH, estimated at 62 mmHg). Chest and abdominal x-rays, as well as cranial and abdominal ultrasounds, showed no abnormalities.

### 2.3 Genetic testing and diagnosis

To elucidate the underlying etiology, trio-WES was performed. The results revealed that the patient carried two heterozygous pathogenic variants in the KLF1 gene: c.519_525dup (p.Gly176ArgfsTer179) and c.1012C > A (p.Pro338Thr). Genetic analysis confirmed that the mother was a heterozygous carrier of c.519_525dup, while the father was a heterozygous carrier of c.1012C > A (p.Pro338Thr). The proband inherited each pathogenic variant from one parent, forming a compound heterozygous state.

This specific variant combination met the pathogenicity criteria outlined by the American College of Medical Genetics and Genomics (ACMG) guidelines. Specifically, c.519_525dup is a null (amorphic) variant that results in a truncated protein lacking the DNA-binding domain, whereas c.1012C > A is a hypomorphic variant. Together, they form a compound heterozygous mutation. The patient was definitively diagnosed with congenital non-spherocytic hemolytic anemia induced by compound heterozygous variants in the KLF1 gene.

### 2.4 Treatment

Given severe anemia, hypoxemia and pulmonary hypertension, the neonate received invasive mechanical ventilation for respiratory support. Multiple packed red blood cell transfusions were administered to correct severe anemia. Inotropic agents were used for hemodynamic stabilization. Inhaled nitric oxide was applied in this case, and pulmonary hypertension improved after anemia correction and respiratory support. No serious adverse events related to transfusion were observed (Table 2).

**Table 2:** Timeline of key clinical events and interventions.

Time	Key clinical events	Interventions
Birth–10 h	Severe pallor, respiratory distress, severe anemia	RBC transfusion, invasive ventilation at outside hospital
10–12 h	Transferred to our NICU. Echocardiography demonstrated a 7- mm PDA and PAH (62 mmHg), with a greater than 10% difference in pre- and post-ductal pulse oximetry.	Mechanical ventilation, hemodynamic monitoring, RBC transfusion, Umbilical artery and venous catheterization (UAC and UVC), inhaled nitric oxide
24–72 h	Progressive hyperbilirubinemia evolving to direct hyperbilirubinemia	Initial phototherapy for jaundice, serial laboratory monitoring
6 d	Improved respiratory status with strong spontaneous breathing; clinical stabilization	Weaning of invasive ventilation
13 d	Persistently elevated ALT and TBA	Oral administration of ursodeoxycholic acid and glucuronolactone
Discharge	Stable without pulmonary hypertension	Discharge home; arrange follow-up

PAH, pulmonary arterial hypertension; PDA, patent ductus arteriosus; RBC, red blood cell; UAC, umbilical artery catheter; UVC, umbilical venous catheter; ALT, alanine aminotransferase; TBA, total bile acid.

### 2.5 Follow-up and genetic counseling

The infant was discharged after clinical stabilization. Twenty-four-month outpatient follow-up confirmed persistent transfusion-dependent anemia. No episode of pulmonary hypertension was noted after discharge. Trio-based genetic testing enabled precise genetic counseling for the parents. Since each parent carries one pathogenic KLF1 variant, this condition follows an autosomal recessive inheritance pattern. For future pregnancies, the recurrence risk is 25% for a fetus inheriting both pathogenic variants. Prenatal genetic diagnosis was therefore offered. The family received psychological support and regular hematology follow-up recommendations.

## 3 Discussion

### 3.1 Genotype-phenotype correlation

The patient's genotype consists of a complete loss-of-function null variant (Class 3) and a partially functional hypomorphic variant (Class 2),according to the functional classification system for *KLF1* variants proposed by Perkins et al. (9). Class 2 represents hypomorphic variants retaining partial residual protein function; Class 3 corresponds to null loss-of-function variants with abolished protein activity. Importantly, Class 4 variants (e.g., p.Glu325Lys, c.973G > A) are gain-of-function dominant variants that can produce severe anemia even in the heterozygous state, which is distinct from the autosomal-recessive compound-heterozygous mechanism observed in our patient (9). The discovery of these compound heterozygous *KLF1* variants—c.519_525dup (p.Gly176ArgfsTer179) and c.1012C > A (p.Pro338Thr)—provides a definitive molecular pathological explanation for the patient's critical clinical phenotype. It also underscores the clinical significance of *KLF1* mutations as an infrequent causative factor for congenital dyserythropoietic anemia (CDA) or Diamond-Blackfan anemia (DBA)-like presentations.

Literature analysis reveals that this compound heterozygous paradigm is predominant among neonatal cases, accounting for 83.3% of presentations (3). The specific combination of p.Gly176ArgfsTer179 and p.Pro338Thr observed in this infant has been previously reported; this particular genotype is strongly associated with severe prenatal cardiomegaly, hydrops fetalis, and severe postnatal transfusion dependence (5). In contrast, fetal cases frequently involve dominant pathogenic variants, whereas childhood cases are characterized primarily by single or homozygous hypomorphic variants. This highlights the varying tolerance thresholds for *KLF1* functional defects across different developmental stages (7, 8).

From an inheritance perspective, the compound heterozygous mutations (resulting in a frameshift truncation and a missense alteration, respectively) conform to an autosomal recessive inheritance pattern that causes a profound loss of *KLF1* function. This aligns with the findings of Kulczynska-Figurny et al. (4), who noted that severe anemia can be triggered by *KLF1* mutations. Clinically, the patient's critical presentation highly correlates with the classic phenotype of severe neonatal *KLF1*-related anemia summarized in the literature. Previous reports have indicated that *KLF1*-associated hemolytic anemia in neonates typically manifests as a rapid onset within 72 h of birth, severe anemia requiring emergency transfusions, and life-threatening pulmonary hypertension (7).Ravindranath et al. also emphasized that the clinical course for neonates with severe *KLF1* mutations is often perilous, originating *in utero* or during the early neonatal period. Other terminal neonatal cases driven by severe *KLF1* mutations have similarly presented with severe pulmonary hypertension and multi-system developmental abnormalities, occasionally culminating in neonatal lethality. The presentation of ultra-severe anemia complicated by pulmonary hypertension shortly after birth in this case further reinforces this link. The underlying mechanism is tightly linked to profound tissue hypoxia and acidosis caused by severe anemia, which subsequently triggers pulmonary vasoconstriction and vascular remodeling (10)^.^

### 3.2 Ultra-early onset and developmental vulnerability

The manifestation of ultra-severe anemia within 10 h of birth indicates that erythroid bone marrow failure had already commenced *in utero* (1). The neonatal period represents a critical window during which erythropoiesis switches from fetal hemoglobin (HbF) to adult hemoglobin (HbA)—a biological process strictly regulated by *KLF1* (2). The compound heterozygous mutation in this case caused a severe loss of *KLF1* transcriptional activity, leading to decompensation when the neonate faced the physiological demand for globin chain switching, thereby explaining the ultra-early, critical phenotype (4).

### 3.3 Pulmonary hypertension and tissue hypoxia

Pulmonary arterial hypertension (PAH) served as a critical complication in this case. Its pathogenesis is primarily attributed to tissue hypoxia and metabolic acidosis induced by the ultra-severe anemia, which precipitates severe pulmonary vasoconstriction. Clinically, management for these patients must not only focus on correcting the anemia but also necessitate timely interventions via mechanical ventilation or inhaled nitric oxide (iNO) to lower pulmonary vascular resistance and improve systemic hemodynamics (11).

### 3.4 Atypical hyperbilirubinemia and complications

A remarkable clinical feature in our patient was the development of predominant direct hyperbilirubinemia after 48–72 h of postnatal life, which is an atypical finding for classic *KLF1*-related anemia. Two major interacting mechanismsmay explain this cholestatic pattern. First, massive ineffective erythropoiesis and hemolysis produce bilirubin overload, predisposing to neonatal cholestasis (3). Second, the large right-to-left shunt via the patent ductus arteriosus could induce hepatic congestion and hemodynamic disturbance, aggravating hepatocellular injury. Previous case summaries reported hepatosplenomegaly in many severely affected patients due to extramedullary hematopoiesis (5). In our neonate, hepatomegaly was also noted, without splenomegaly, representing a variable phenotypic expression.

Severe pulmonary hypertension is a well-documented life-threatening complication in neonatal *KLF1*-related disease. Profound tissue hypoxia and metabolic acidosis secondary to ultra-severe anemia trigger pulmonary vasoconstriction. Urgent management includes red-blood-cell transfusion to improve oxygen-carrying capacity, alongside pulmonary vasodilator strategies such as mechanical ventilation and inhaled nitric oxide when indicated (3, 11).

### 3.5 Diagnostic challenges and pitfalls in clinical differentiation

The positive screening result for thalassemia in this neonate was highly confounding. Mechanistically, this represents a classic false-positive result driven by the failure of globin switching due to *KLF1* deficiency, which leads to a sustained, abnormal elevation of HbF (12, 13). Jamwal M et al. reported the first case from the Indian subcontinent where neonatal complications included sepsis, neonatal cholestasis, respiratory distress, and a patent ductus arteriosus alongside severe anemia; notably, laboratory evaluations for plasma hemoglobin, G6PD deficiency, autoimmune hemolytic anemia, *β*-thalassemia, and hereditary spherocytosis were all negative (14).

When clinicians encounter unexplained, severe neonatal hemolysis that is DAT-negative, they must maintain a high index of suspicion for rare genetic etiologies like *KLF1* abnormalities once common causes have been ruled out (15). Timely deployment of whole-exome sequencing (WES) using non-peripheral blood samples (such as buccal mucosal cells) presents the optimal path toward achieving a precision diagnosis (3, 16). Because *KLF1* mutations are relatively more common in thalassemia-endemic regions and can ameliorate the severity of *β*-thalassemia, screening for them has become increasingly vital during the diagnostic workup of unexplained, DAT-negative congenital hemolytic anemia (17).

Consequently, in neonates presenting with severe anemia that is DAT-negative, if conventional thalassemia genetic testing fails to explain the clinical phenotype, *KLF1* variants must be highly suspected (14, 18).

### 3.6 Long-term management and prognosis

Severely affected infants are typically profoundly transfusion-dependent during infancy and require rigorous monitoring of iron metabolism to prevent secondary iron overload (12). Although bone marrow compensation may improve with age in some patients, thereby reducing transfusion requirements (19), allogeneic hematopoietic stem cell transplantation (HSCT) currently represents the definitive curative option for those with persistent, severe transfusion dependence (3).

Of note, loss-of-function *KLF1* variants have been reported to trigger re-expression of embryonic globin genes besides elevated fetal-hemoglobin levels, which serves as an important molecular signature of disturbed erythroid maturation (20). Unfortunately, globin-expression profiling including embryonic globin analysis could not be performed in the present retrospective case report because no residual biological samples were available. As this is a single-case retrospective report, general inferences cannot be drawn from this individual patient. Despite these limitations, our observation provides important real-world phenotypic data for this rare compound-heterozygous *KLF1* genotype and highlights the clinical triad of severe neonatal anemia, direct hyperbilirubinemia, and pulmonary hypertension to alert front-line clinicians. Detection of embryonic- and fetal-globin signatures would provide valuable supplementary evidence for *KLF1*-related anemia in future prospective studies (20).

## 4 Conclusion

*KLF1* gene mutations represent a critical etiology of unexplained congenital neonatal anemia. The clinical triad of immediate postnatal severe anemia, rapidly progressive direct hyperbilirubinemia, and pulmonary hypertension serves as a vital diagnostic indicator. Timely genetic diagnosis is paramount to avoiding redundant clinical investigations, initiating targeted management, and providing accurate familial genetic counseling.

*KLF1* gene variations are an important factor underlying unexplained severe neonatal anemia and non-immune hydrops fetalis. When a neonate presents with ultra-early onset severe hemolytic anemia accompanied by pulmonary hypertension or unexplained direct bilirubin elevation, WES should be initiated promptly to secure a molecular diagnosis, thereby guiding individualized therapy and reproductive counseling.

## Data Availability

The datasets presented in this study can be found in online repositories. The names of the repository/repositories and accession number(s) can be found in the article/Supplementary Material.
